# Supplementary material for: Olprinone, a Selective Phosphodiesterase III Inhibitor, Has Protective Effects in a Septic Rat Model after Partial Hepatectomy and Primary Rat Hepatocyte
Source: Int J Mol Sci. 2024 Jun 29;25(13):7189. doi: 10.3390/ijms25137189 (PMC11241400; doi:10.3390/ijms25137189)
Supplement: Supplementary file 1 [file ijms-25-07189-s001.zip › ijms-2989946-supplementary.pptx]

## Slide 1
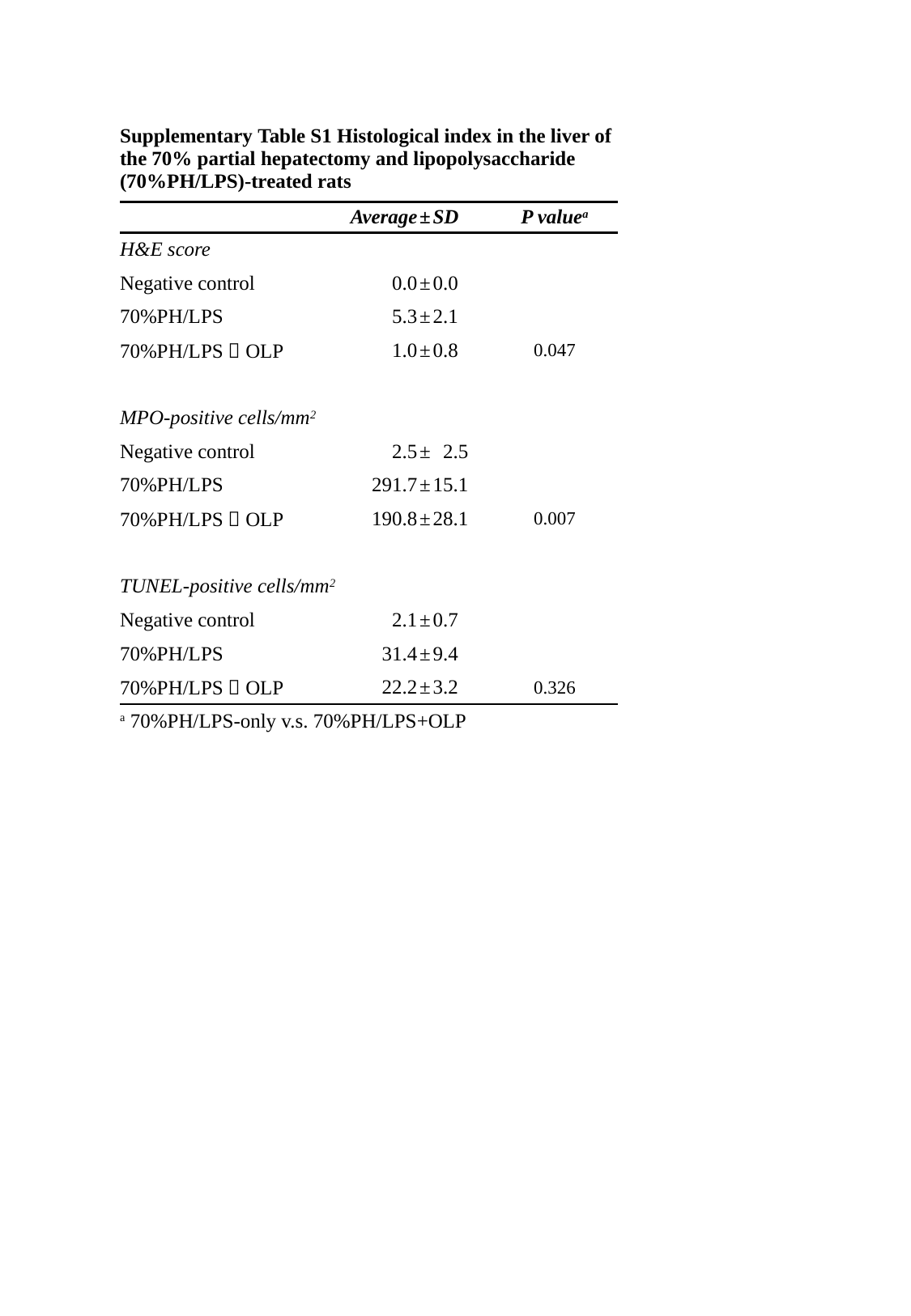

| Supplementary Table S1 Histological index in the liver of the 70% partial hepatectomy and lipopolysaccharide (70%PH/LPS)-treated rats ​ | | | | |
| --- | --- | --- | --- | --- |
| | Average | ± | SD | P valuea |
| H&E score | | | | |
| Negative control | 0.0 | ± | 0.0 | |
| 70%PH/LPS | 5.3 | ± | 2.1 | |
| 70%PH/LPS＋OLP | 1.0 | ± | 0.8 | 0.047 |
| | | | | |
| MPO-positive cells/mm2 | | | | |
| Negative control | 2.5 | ± | 2.5 | |
| 70%PH/LPS | 291.7 | ± | 15.1 | |
| 70%PH/LPS＋OLP | 190.8 | ± | 28.1 | 0.007 |
| | | | | |
| TUNEL-positive cells/mm2 | | | | |
| Negative control | 2.1 | ± | 0.7 | |
| 70%PH/LPS | 31.4 | ± | 9.4 | |
| 70%PH/LPS＋OLP | 22.2 | ± | 3.2 | 0.326 |
| a 70%PH/LPS-only v.s. 70%PH/LPS+OLP | | | | |

## Slide 2
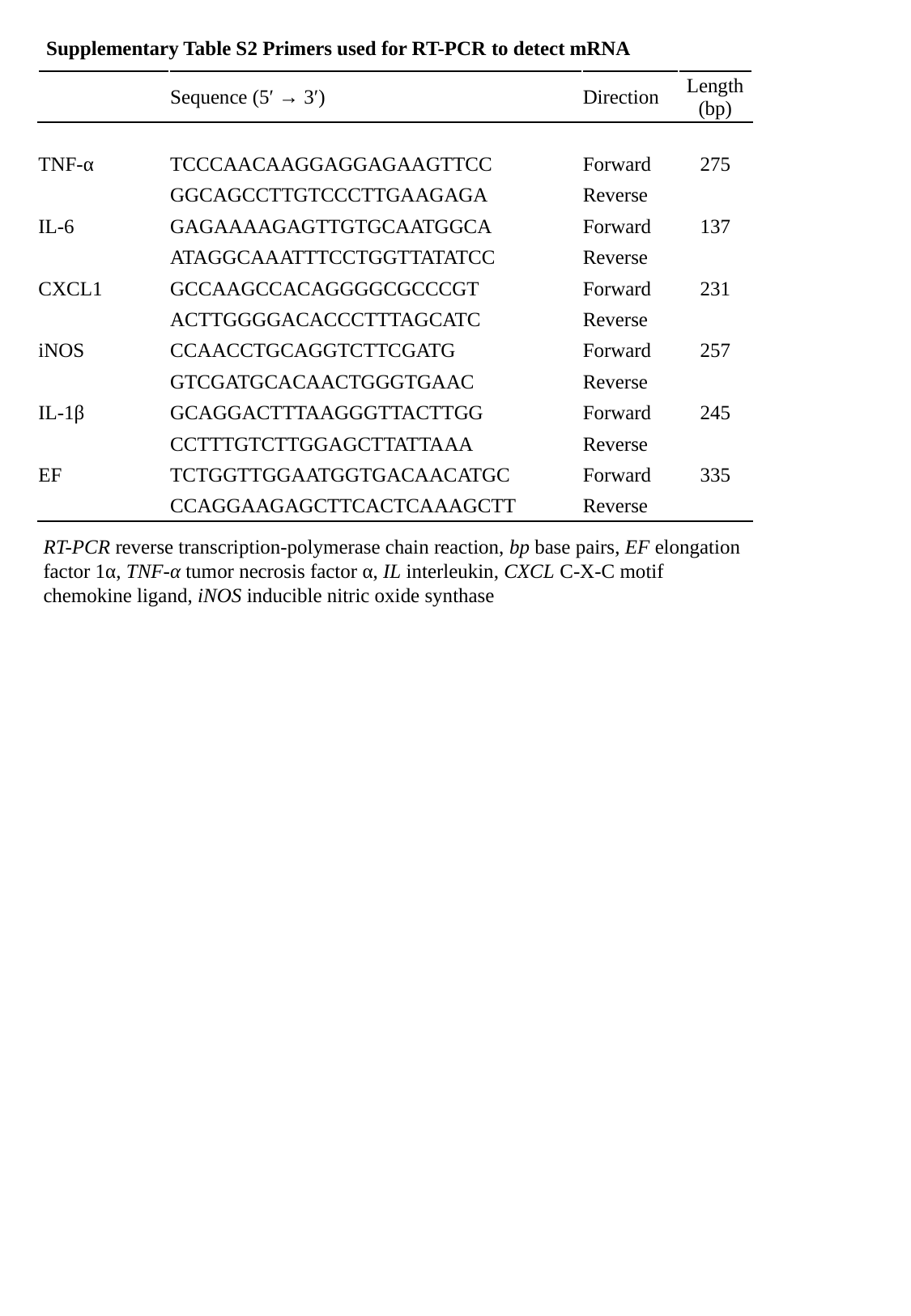

Supplementary Table S2 Primers used for RT-PCR to detect mRNA
| | Sequence (5′ → 3′) | Direction | Length (bp) |
| --- | --- | --- | --- |
| | | | |
| TNF-α | TCCCAACAAGGAGGAGAAGTTCC | Forward | 275 |
| | GGCAGCCTTGTCCCTTGAAGAGA | Reverse | |
| IL-6 | GAGAAAAGAGTTGTGCAATGGCA | Forward | 137 |
| | ATAGGCAAATTTCCTGGTTATATCC ​ | Reverse | |
| CXCL1 | GCCAAGCCACAGGGGCGCCCGT | Forward | 231 |
| | ACTTGGGGACACCCTTTAGCATC | Reverse | |
| iNOS | CCAACCTGCAGGTCTTCGATG | Forward | 257 |
| | GTCGATGCACAACTGGGTGAAC | Reverse | |
| IL-1β | GCAGGACTTTAAGGGTTACTTGG | Forward | 245 |
| | CCTTTGTCTTGGAGCTTATTAAA | Reverse | |
| EF | TCTGGTTGGAATGGTGACAACATGC | Forward | 335 |
| | CCAGGAAGAGCTTCACTCAAAGCTT | Reverse | |
RT-PCR reverse transcription-polymerase chain reaction, bp base pairs, EF elongation factor 1α, TNF-α tumor necrosis factor α, IL interleukin, CXCL C-X-C motif chemokine ligand, iNOS inducible nitric oxide synthase
